# Instantaneous Wave-Free Ratio for the Assessment of Intermediate Left Main Coronary Artery Stenosis: Correlations With Fractional Flow Reserve/Intravascular Ultrasound and Prognostic Implications: The iLITRO-EPIC07 Study

**DOI:** 10.1161/CIRCINTERVENTIONS.122.012328

**Published:** 2022-09-16

**Authors:** Oriol Rodriguez-Leor, José María de la Torre Hernández, Tamara García-Camarero, Bruno García del Blanco, Ramón López-Palop, Eduard Fernández-Nofrerías, Carlos Cuellas Ramón, Marcelo Jiménez-Kockar, Jesús Jiménez-Mazuecos, Francisco Fernández Salinas, Josep Gómez-Lara, Salvatore Brugaletta, Fernando Alfonso, Ricardo Palma, Antonio E. Gómez-Menchero, Raúl Millán, David Tejada Ponce, José Antonio Linares Vicente, Soledad Ojeda, Eduardo Pinar, Estefanía Fernández-Pelegrina, Francisco J. Morales-Ponce, Ana Belén Cid-Álvarez, Juan Carlos Rama-Merchan, Eduardo Molina Navarro, Javier Escaned, Armando Pérez de Prado

**Affiliations:** Institut del Cor, Hospital Universitari Germans Trias i Pujol, Badalona, Spain (O.R.-L., E.F.-N.).; Centro de Investigación Biomédica en Red de Enfermedades Cardiovasculares (CIBERCV), Spain (O.R.-L., E.F.-N., A.B.C.-A.).; Institut de Recerca en Ciències de la Salut Germans Trias i Pujol, Badalona, Spain (O.R.-L., E.F.-N.).; Cardiology Department, Hospital Universitario Marqués de Valdecilla, Santander, Spain (J.M.d.l.T.H., T.G.-C.).; Instituto de Investigación Marqués de Valdecilla (IDIVAL), Santander, Spain (J.M.d.l.T.H., T.G.-C.).; Cardiology Department, Hospital Universitari Vall d’Hebron, Barcelona, Spain (B.G.d.B., R.P.).; Cardiology Department, Hospital Virgen de la Arrixaca, Murcia, Spain (R.L.-P., E.P.).; Servicio de Cardiología, Hospital Universitario de León, Spain (C.C.R., A.P.d.P.).; Cardiology Department, Hospital de la Santa Creu i Sant Pau, Barcelona, Spain (M.J.-K., E.F.-P.).; Servicio de Cardiología, Complejo Hospitalario Universitario de Albacete, Spain (J.J.-M.).; Cardiology Department, Hospital Universitari Joan XXIII, Tarragona, Spain (F.F.S.).; Cardiology Department, Hospital Universitari de Bellvitge, L’Hospital de Llobregat, Spain (J.G.-L.).; Cardiology Department, Hospital Clínic, Barcelona, Spain (S.B.).; Institut d’Investigacions Biomèdiques August Pi i Sunyer (IDIBAPS), Barcelona, Spain (S.B.).; University of Barcelona, Spain (S.B.).; Cardiology Department, Hospital de la Princesa, Madrid, Spain (F.A.).; Cardiology Department, Hospital Universitario Juan Ramon Jiménez, Huelva, Spain (A.E.G.-M.).; Cardiology Department, Hospital del Mar, Barcelona, Spain (R.M.).; Cardiology Department, Hospital General Universitario de Castellón, Castellón de la Plana, Spain (D.T.P.).; Cardiology Department, Hospital Clínico Universitario Lozano Blesa, Zaragoza, Spain (J.A.L.V.).; Division of Interventional Cardiology, Hospital Universitario Reina Sofía, Córdoba, Spain (S.O.).; University of Cordoba, Cordoba, Maimonides Institute for Research in Biomedicine of Cordoba (IMIBIC), Spain (S.O.) Cardiology Department, Hospital Universitario de Puerto Real, Spain (F.J.M.-P.).; Servicio de Cardiología, Hospital Clínico Universitario de Santiago, Santiago de Compostela, Spain (A.B.C.-A.).; Cardiology Department, Hospital Universitario Virgen de las Nieves, Granada, Spain (J.C.R.-M.).; Cardiology Department, Hospital de Mérida, Extremadura, Spain (E.M.N.).; Cardiology Department, Hospital Clínico San Carlos, Madrid, Spain (J.E.).; Instituto de Investigación Sanitaria Hospital Clínico San Carlos (IdSSC), Madrid, Spain (J.E.).; Universidad Complutense de Madrid, Spain (J.E.).

**Keywords:** coronary artery disease, left main coronary artery disease, ultrasound imaging

## Abstract

There is little information available on agreement between fractional flow reserve (FFR) and instantaneous wave-free ratio (iFR) in left main coronary artery (LMCA) intermediate stenosis. Besides, several meta-analyses support the use of FFR to guide LMCA revascularization, but limited information is available on iFR in this setting. Our aims were to establish the concordance between FFR and iFR in intermediate LMCA lesions, to evaluate with intravascular ultrasound (IVUS) in cases of FFR/iFR discordance, and to prospectively validate the safety of deferring revascularization based on a hybrid decision-making strategy combining iFR and IVUS.

**Methods:**

Prospective, observational, multicenter registry with 300 consecutive patients with intermediate LMCA stenosis who underwent FFR and iFR and, in case of discordance, IVUS and minimal lumen area measurements. Primary clinical end point was a composite of cardiovascular death, LMCA lesion-related nonfatal myocardial infarction, or unplanned LMCA revascularization.

**Results:**

FFR and iFR had an agreement of 80% (both positive in 67 and both negative in 167 patients); in case of disagreement (31 FFR+/iFR– and 29 FFR−/iFR+) minimal lumen area was ≥6 mm^2^ in 8.7% of patients with FFR+ and 14.6% with iFR+. Among the 300 patients, 105 (35%) underwent revascularization and 181 (60%) were deferred according to iFR and IVUS. At a median follow-up of 20 months, major adverse cardiac events incidence was 8.3% in the defer group and 13.3% in the revascularization group (hazard ratio, 0.71 [95% CI 0.30–1.72]; *P*=0.45).

**Conclusions:**

In patients with intermediate LMCA stenosis, a physiology-guided treatment decision is feasible either with FFR or iFR with moderate concordance between both indices. In case of disagreement, the use of IVUS may be useful to indicate revascularization. Deferral of revascularization based on iFR appears to be safe in terms of major adverse cardiac events.

**Registration:**

URL: https://www.clinicaltrials.gov; Unique identifier: NCT03767621.

**What is Known**

- Angiographic assessment of a left main coronary artery (LMCA) stenosis is often difficult and unreliable, so it is recommended to confirm lesion severity with intracoronary imaging or physiology. The LITRO trial (Prospective Use of an Intravascular Ultrasound-Derived Minimum Lumen Area Cut-Off Value in the Assessment of Intermediate Left Main Coronary Artery Lesions) demonstrated that deferred revascularization when intravascular ultrasound (IVUS) minimal lumen area was superior or equal to 6 mm^2^ was safe. Previous studies have shown that instantaneous wave-free ratio (iFR)-guided coronary revascularization was not inferior to fractional flow reserve (FFR)-guided revascularization with respect to the risk of major cardiac events, but these studies excluded patients with LMCA stenosis.
- Agreement between FFR and iFR in non-LMCA lesions is moderate, and there is little information on agreement in LMCA. Several meta-analyses support the use of FFR to guide left main coronary artery revascularization, but limited information is available on iFR in this setting.

**What the Study Adds**

- We have shown that agreement between FFR and iFR in LMCA stenosis is moderate, but with a good correlation between the 2 indices, and that it was better when measured in the left circumflex than when measured in the left anterior descending coronary artery.
- In case of discordance, when FFR was positive IVUS showed an minimal lumen area <6 mm^2^ in 69% of cases, whereas when iFR was positive, IVUS showed an minimal lumen area <6 mm^2^ in 40% of cases.
- In patients with intermediate LMCA stenosis in whom revascularization was deferred on the basis of iFR results (combined with IVUS in case of FFR-iFR discordance), the incidence of major adverse cardiac events during follow-up was low with no differences compared with patients treated with revascularization, whereas the incidence of myocardial infarction related to LMCA lesion tended to be lower in patients with deferred revascularization.

Due to the limitations of the coronary angiogram in assessing functional relevance of intermediate left main coronary artery (LMCA) stenosis,^1–3^ invasive image modalities such as intravascular ultrasound (IVUS) or optical coherence tomography as well as invasive functional techniques (fractional flow reserve [FFR] or instantaneous wave-free ratio [iFR]) are recommended to guide revascularization decision.^4^ The LITRO study (Prospective Use of an Intravascular Ultrasound-Derived Minimum Lumen Area Cut-Off Value in the Assessment of Intermediate Left Main Coronary Artery Lesions) showed that a minimal lumen area (MLA) of 6 mm^2^ or more was a safe value for deferring revascularization.^5^ Use of FFR in this setting is supported by a limited number of nonrandomized studies that confirmed that deferral of LM stenosis revascularization when FFR shows nonischemic values is safe, with similar or better patient outcomes than patients undergoing treatment based on abnormal FFR values.^6,7^ Similarly to FFR, a nonrandomized study on the value of iFR in the decision-making process regarding LMCA stenosis reported that LMCA revascularization deferral based on nonischemic iFR values is safe.^8^ Because none of the above-mentioned studies in LMCA stenoses performed measurements with both FFR and iFR, the frequency and meaning of discordant values between both indices remains unknown, although studies looking into the overall discordance in functional stenosis classification with FFR and iFR suggest that it may be higher in LMCA than in other coronary locations.^9,10^ Thus, the frequency and clinical significance of discordant FFR and iFR values in LMCA stenoses remains unknown.

The objective of the iLITRO-EPIC 07 study (Concordance Between FFR and iFR for the Assessment of Intermediate Lesions in the Left Main Coronary Artery: A Prospective Validation of a Default Value for iFR) was to prospectively assess the degree of agreement between FFR and iFR in terms of functional classification of intermediate LMCA stenosis, using IVUS evaluation in cases of disagreement between both physiology indices. It also was aimed to assess the safety of a hybrid decision-making strategy combining iFR and IVUS in intermediate LMCA stenoses.

## METHODS

### Study Design

The design of the iLITRO-EPIC07 study has been described previously^11^ and is reported as supplementary material. Briefly, it is a prospective, observational, multicenter registry that enrolled consecutive patients with intermediate LMCA lesions (visual estimation 25–65% diameter stenosis^5^) in 33 centers between November 2018 and November 2021. Patients underwent both FFR and iFR measurements distal to LMCA stenosis. The study protocol recommended a specific physiology and intravascular imaging-based algorithm to guide intermediate LMCA stenosis management:

1. In patients with nonsignificant FFR and iFR values distal to LMCA stenosis (>0.80 and >0.89, respectively), optimal medical treatment plus deferral revascularization was recommended. Other lesions outside LMCA with revascularization indication should be treated by percutaneous coronary intervention (PCI). By protocol, IVUS was recommended whenever possible.

2. In patients with significant FFR and iFR values distal to LMCA stenosis (≤0.80 and ≤0.89, respectively), revascularization of the LMCA lesion was recommended (either with PCI or coronary artery bypass grafting [CABG]). Other lesions outside LMCA with revascularization indication should be treated by PCI or CABG. By protocol, IVUS was recommended whenever possible.

3. In case of discrepancy between FFR and iFR distal to LMCA (>0.80 and ≤0.89 or ≤0.80 and >0.89, respectively), IVUS was performed to decide on revascularization; if MLA<6 mm^2^ revascularization of the LMCA lesion was recommended (PCI or CABG) and in case of MLA≥6 mm^2^, clinical follow-up without LMCA lesion revascularization was recommended.

The study was approved by the reference ethics committee and notified to the local ethics committee of all participant centers. The study was registered in Clinicaltrials.gov with registration number NCT03767621. Only devices with CE (Conformité Européenne) marking were used, and only for the indications already approved. The study observed the principles established by the Declaration of Helsinki. All patients gave their written informed consent prior to participation in the study. The data that support the findings of this study are available from the corresponding author upon reasonable request.

### Study Population

Patients with suspected or confirmed ischemic heart disease showing intermediate LMCA lesion in the coronary angiography (visual estimation between 25% and 60% diameter stenosis) were eligible. Inclusion and exclusion criteria have been published previously^11^ and are reported as supplementary material.

In case of severe stenosis at left anterior descending coronary artery (LAD) or left circumflex coronary artery (LCX), LMCA lesion was assessed after PCI of these lesions to avoid artifact in measurements (especially in FFR), provided that the investigators considered that no CABG indication was considered to exist in case of significant LMCA stenosis.

Technical aspects regarding intracoronary pressure wire measurements have been previously reported^11^ and are resumed in supplementary material.

A 12-months and 5-year follow-up were scheduled. Follow-up information was prospectively collected in on-site visits and if required by reviewing clinical reports or by telephone, in all cases. The indication for repeated catheterization was driven clinically and was decided by the clinical cardiologists.

### Objectives

The iLITRO-EPIC07 study had 2 primary objectives: (1) to establish the concordance between FFR and iFR in intermediate LMCA lesions to defer revascularization, with cut-off values >0.80 (with IV adenosine) for FFR and >0.89 for iFR; and (2) to prospectively assess the safety of a hybrid decision-making strategy for deferring revascularization in patients with intermediate LMCA lesion, based on an iFR cut-off value >0.89 and, in case of FFR and iFR discordance, an MLA≥6 mm^2^.

Primary end point was a composite of cardiovascular death, LMCA lesion-related nonfatal myocardial infarction or unplanned LMCA revascularization at maximum follow-up. All clinical events were adjudicated by an independent clinical event adjudication committee that was blinded to FFR, iFR, and IVUS results.

### Statistical Analysis

Demographic, clinical, hemodynamic, and procedural data are presented for the entire group. Continuous variables are expressed as mean and SD. Categorical variables are expressed as frequencies and percentages. The data obtained are analyzed using the unilateral ANOVA or pairwise *t* test for continuous variables and χ^2^ test for categorical variables. The major adverse cardiac events (MACE)-free survival data were represented and analyzed using Kaplan-Meier curves and Cox regression analysis. For the first study objective, concordance between functional and imaging techniques have been conducted using Cohen’s kappa coefficient. Also, sensitivity, specificity, positive and negative predictive values, and the area under the receiver operating characteristic curve are estimated. Given that iFR values do not follow a normal distribution, Spearman test has been used to establish correlation between FFR and iFR. For the secondary study objective, clinical outcomes have been reported separately between patients in whom LMCA revascularization was based on the iFR values, as per protocol, and those who were not. A *P* value of 0.05 is considered to set statistical significance. All analyses are performed with the use Stata 15.0 (Stata Corp, College Station, TX).

## Results

The study enrolled 300 consecutive patients with intermediate LMCA lesions (visual estimation 25–65% diameter stenosis). Both LMCA FFR and iFR were recorded in 293 (98%) patients (from LAD in 291 patients and from LCX in 257 patients) while in 6 patients only iFR from LAD and/or LCX was recorded.

### FFR/iFR Concordance

**Figure 1.**
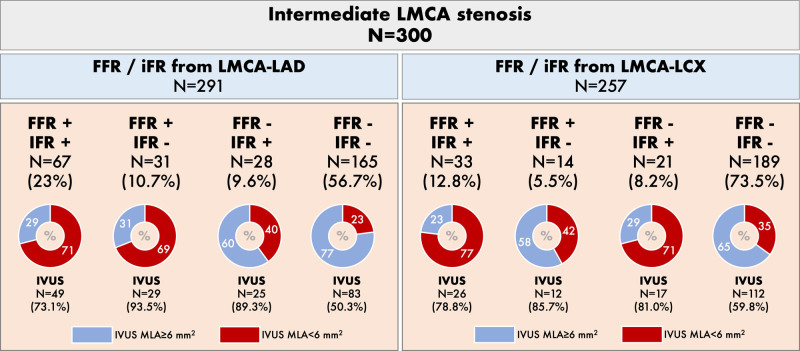
Patient classification according to the concordance between fractional flow reserve (FFR) and instantaneous wave-free ratio (iFR) as well as intravascular ultrasound (IVUS) significance. FFR and iFR were measured from left anterior descending coronary artery (LAD) in 291 patients and from left circumflex coronary artery (LCX) in 257 patients. There were 3 patients with measurements from LCX but not from the LAD, and 6 patients only with iFR measurement. LMCA indicates left main coronary artery; and MLA, minimal lumen area.

**Figure 2.**
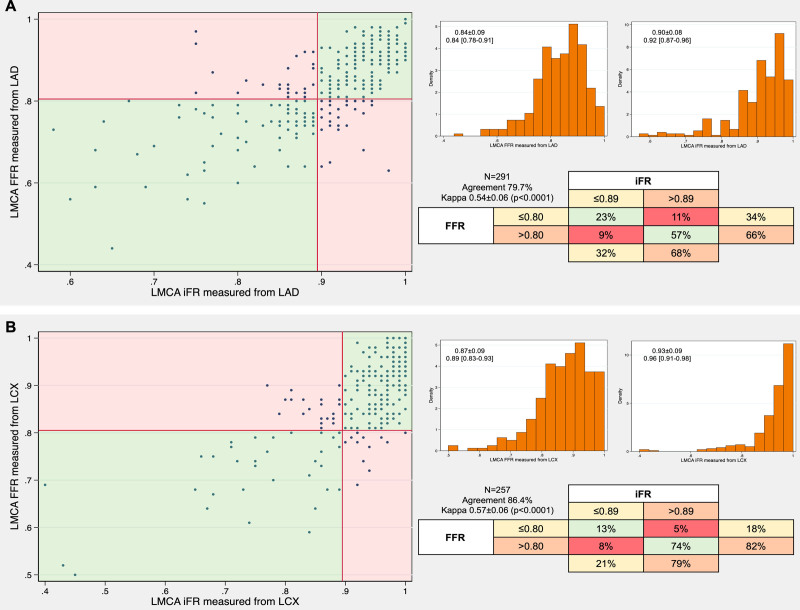
Fractional flow reserve (FFR) and instantaneous wave-free ratio (iFR) correlation, receiver operating curve and values distribution when measured from left anterior descending coronary artery (LAD) and left circumflex coronary artery (LCX). **A**, Measurements from LAD; (**B**) measurements from LCX. LMCA indicates left main coronary artery.

Figure 1 shows the 4 groups according to concordant and discordant FFR and iFR measurements, performed either from the LAD or LCX vessels, as well and the agreement with IVUS MLA significance (cut-off≥6 mm^2^). Table 1 shows clinical, angiographical, physiological, and IVUS characteristics, according to FFR and iFR concordance. Figure 2 shows FFR and iFR correlation and values distribution when measured from LAD and LCX.

**Table 1.**
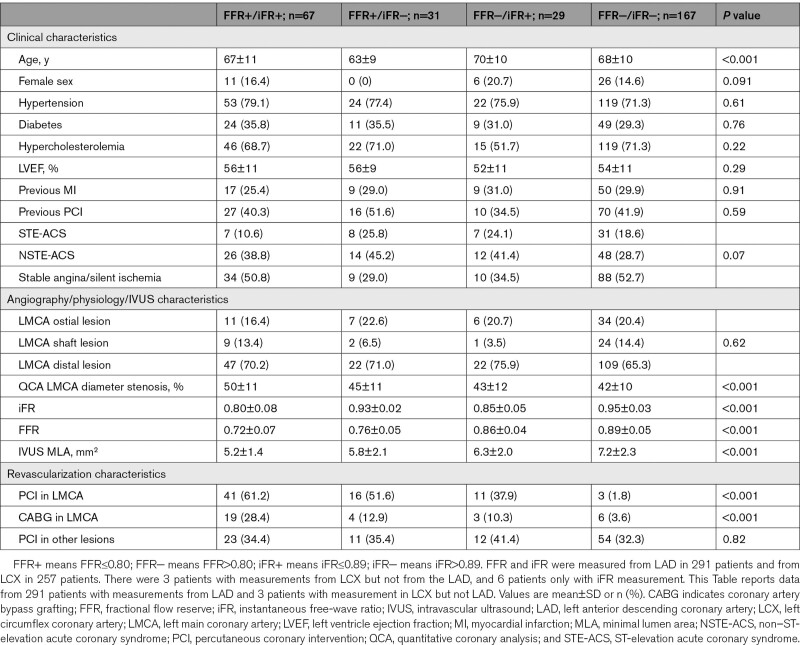
Clinical, Angiographical, Physiological, and IVUS Characteristics According to FFR and iFR Concordance

Concordance between FFR and iFR values was worse when measurements were performed from the LAD than from the LCX (79.7% of agreement with correlation *r*=0.70 and κ=0.54±0.06 versus 86.4% agreement with correlation *r*=0.65 and κ=0.57±0.06, respectively). According to physiologic indices, revascularization deferral of LMCA would take place in 67.4% for iFR and 66.3% for FFR when indices measured from LAD (1.1% more deferral according to iFR). Alternatively, revascularization deferral would take place in 79.0% and 81.7% of patients according to iFR and FFR values performed in the LCX (2.7% more deferral according to FFR).

Figure S1 shows FFR and iFR correlation considering the grey zone for both indices (0.75–0.80 for FFR and 0.86–0.92 for iFR). With this approach, only 4% of cases were discordant when measured from LAD and 3% when measured from LCX.

### IVUS Concordance With FFR and iFR

IVUS imaging was performed in 192 patients (64%). According to the study protocol, 55 out of the 60 patients (91.7%) with discordant FFR and iFR values were imaged with IVUS.

**Figure 3.**
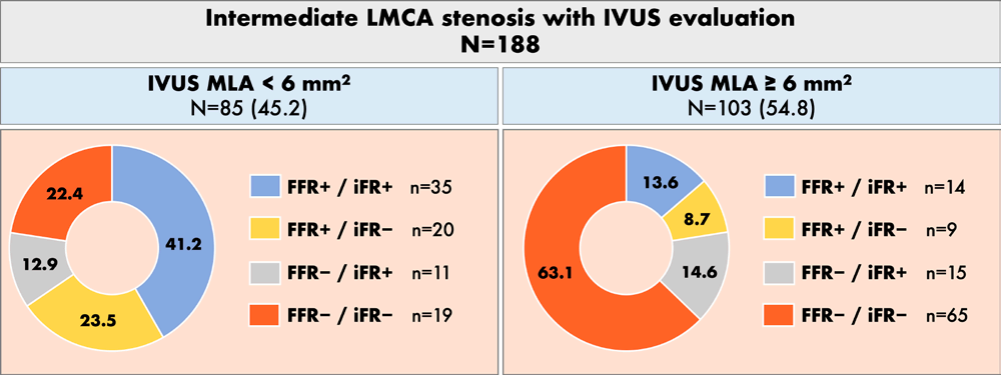
Concordance between fractional flow reserve (FFR) and instantaneous wave-free ratio (iFR) according to a minimal lumen area (MLA) cutoff of 6 mm^2^ in patients with intravascular ultrasound (IVUS) evaluation. This analysis was performed in 188 patients with MLA, FFR, and iFR measurements (in 6 patients with IVUS study only iFR was measured). LMCA indicates left main coronary artery.

Figure 3 shows the FFR and iFR values obtained in the LAD according to the IVUS MLA (<6 mm^2^ or ≥6 mm^2^). When MLA was <6 mm^2^ (n=85, 42.5%), 30 patients (35.3%) had FFR>0.80 and 39 patients (45.9%) had iFR>0.89. When MLA was ≥6 mm^2^ (n=103, 54.8%), 23 patients (22.3%) had FFR≤0.80 and 29 patients (28.2%) had iFR≤0.89.

An MLA cut point of 6.0 mm^2^ had the highest sensitivity, specificity, and predictive accuracy to predict an FFR>0.80 measured from LAD (sensibility 73%, specificity 71%, predictive accuracy 72%), as well as to predict an iFR>0.89 measured from LAD (sensitivity 66%, specificity 60%, predictive accuracy 63%; Figure S2).

### Treatment Decision According to iFR, FFR, and MLA

Treatment decision was LMCA revascularization in 105 patients (35%), and LMCA revascularization defer according to the protocol recommendation in 181 patients (60.3%).

**Figure 4.**
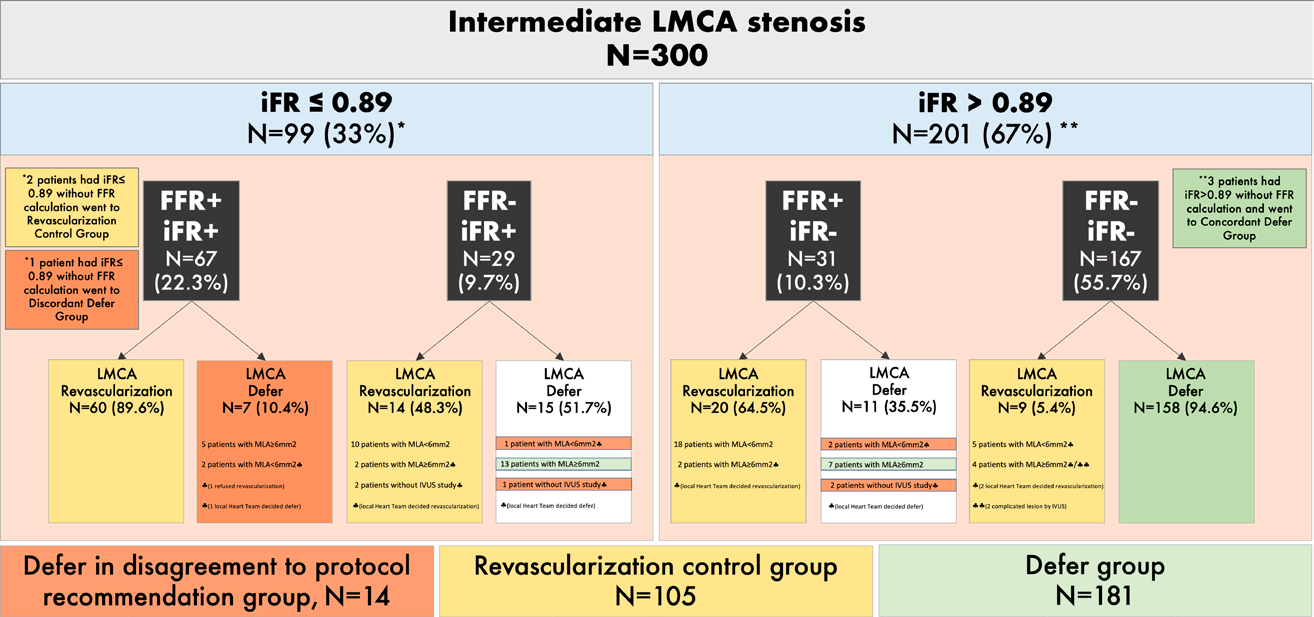
Treatment decision according to instantaneous wave-free ratio (iFR), fractional flow reserve (FFR), and minimal lumen area (MLA) values. In patients with discordant FFR and iFR, protocol recommended to perform intravascular ultrasound (IVUS) and decide revascularization if MLA<6 mm^2^, but local heart team had the final decision. LMCA indicates left main coronary artery.

Figure 4 shows treatment decision according to iFR, FFR, and MLA values. Except for 1 patient with positive FFR, iFR, and MLA who refused revascularization, decision of treatment was based on local heart team recommendation in all cases.

There were 15 patients (5%) with LMCA revascularization and 14 patients (4.7%) with revascularization deferral in which decision disagreed to protocol recommendation. Table S1 summarizes the reasons for not following the protocol recommendations.

Table 2 shows clinical, angiographical, physiological, and IVUS characteristics according to LMCA stenosis management decision (revascularization or defer according to protocol recommendation).

**Table 2.**
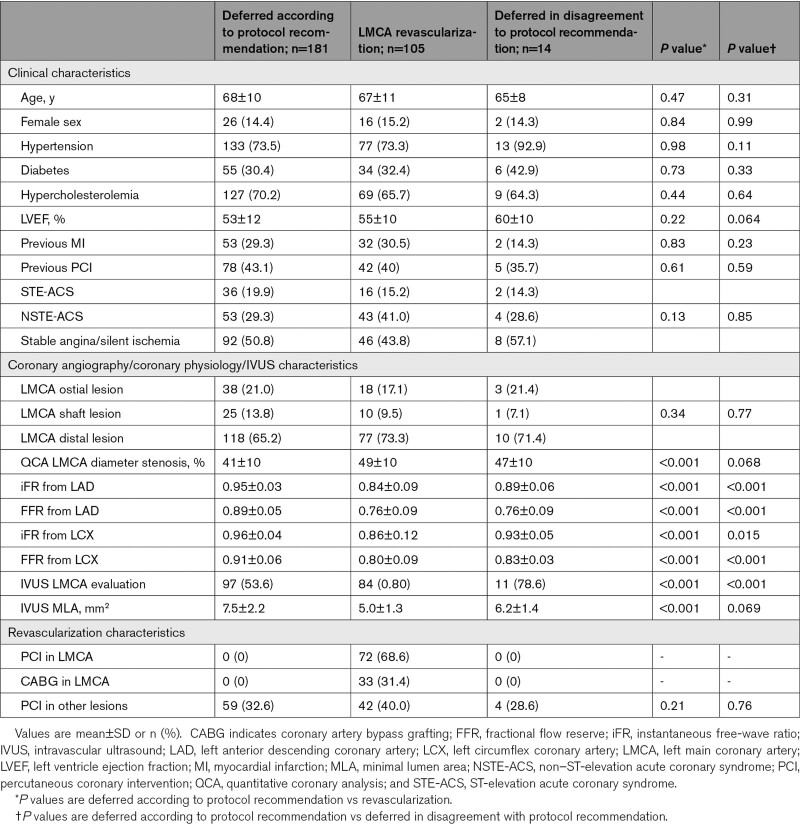
Clinical, Angiographical, Physiological, and IVUS Characteristics According to the LMCA Stenosis Management Decision

### Safety of Deferring Revascularization Based on a Hybrid Decision-Making Strategy Combining iFR and IVUS

MACE incidence was 8.3% in the defer group and 13.3% in the revascularization group (hazard ratio, 0.71 [95% CI 0.30–1.72]; *P*=0.45). Table 3 shows incidence of different components of MACE in each group.

**Table 3.**
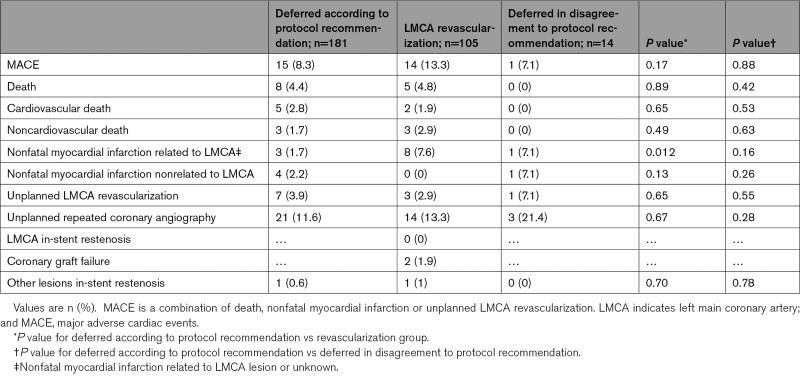
Clinical Outcomes Within 1-Year Follow-Up

Incidence of nonfatal myocardial infarction related to LMCA lesion had a trend to be lower in the concordant defer group compared to revascularization group (1.7% versus 7.6%; hazard ratio, 0.28 [95% CI 0.07–1.11]; *P*=0.06).

Patients who underwent CABG showed higher incidence of subsequent MACE compared to patients treated with PCI (21.2% versus 5.6%; hazard ratio, 8.16 [95% CI 1.69–39.29]; *P*=0.009).

**Figure 5.**
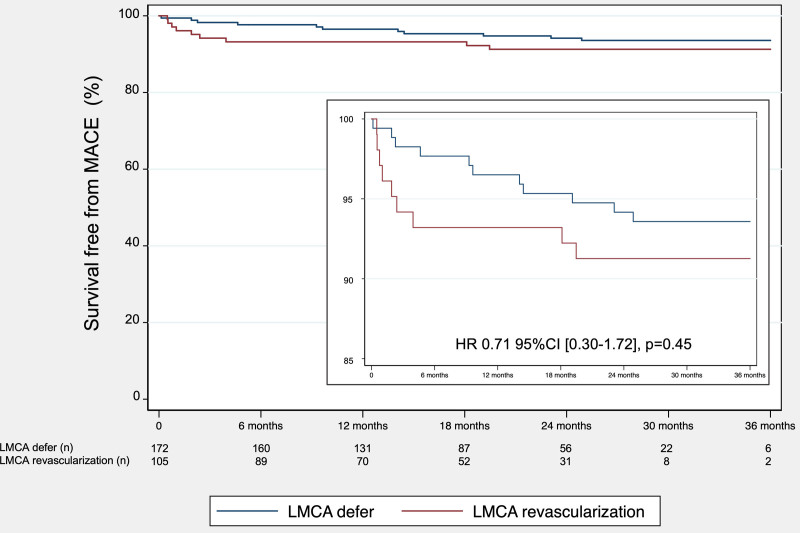
Major adverse cardiac events (MACE) in follow-up. Kaplan-Meier event-free curves showing major cardiac events in the 2 groups. There was no difference between the deferred according to protocol recommendation and revascularized groups. HR indicates hazard ratio; and LMCA, left main coronary artery.

Figure 5 shows MACE-free survival at a median follow-up of 20 months in patients with deferred LMCA revascularization according to the protocol recommendation compared to patients with LMCA revascularization.

## Discussion

To the best of our knowledge, this is the first prospective study investigating the concordance between FFR and iFR in patients with intermediate LMCA stenosis and the first study adding IVUS data for discordant cases. Moreover, this is the first study systematically assessing long-term outcomes in these challenging patients when decision to perform or defer revascularization was based on IVUS, and when FFR and iFR measurements were not concordant.

Our main findings are as follows. First, we have found an 80% of concordance between FFR and iFR to establish revascularization indication when measured from LAD and concordance was better when measured from LCX that from LAD. In case of discordance, when FFR was positive and iFR was negative, IVUS showed an MLA<6 mm^2^ in 69% of cases, while when FFR was negative and iFR was positive, IVUS showed an MLA<6 mm^2^ in 40% of cases. Second, in patients with intermediate LMCA stenosis with discordance between FFR and iFR in whom revascularization was deferred on the basis of IVUS MLA≥6 mm^2^, MACE incidence during follow-up was low without differences compared to patients treated with revascularization. Moreover, incidence of myocardial infarction related to LMCA lesion tend to be lower in patients with revascularization deferred.

### Concordance Between FFR and iFR

Lesion location in LMCA has been described as a predictor of worse concordance between FFR and iFR. Kobayashi et al^9^ compared FFR and iFR in 760 patients, 201 with lesion located in LMCA or proximal LAD, and found less agreement, taking FFR as gold standard, in LMCA or proximal LAD compared to other locations (*r*=0.66 and area under the curve by receiver operating characteristic 0.79). In our study, correlation between FFR and iFR was slightly higher (*r*=0.70 with area under the curve by receiver operating characteristic 0.86); this slight difference can be explained by the different patient clinical characteristics between both studies, with more diabetes and more acute coronary syndrome in iLITRO, and also by the absence of untreated distal severe stenoses in our study. Dérimay et al^10^ described concordance in 587 patients and found that lesions in LMCA or proximal LAD were predictors of negative discordant iFR; in fact, in 150 patients with LMCA and proximal LAD lesions, they described only 51% of concordance. Other predictors of negative discordant iFR were more severe stenosis, younger age, and lower heart rate, while absence of beta-blocker, older age, and less severe stenosis were predictors of a positive discordant iFR. In LAD, but not in other locations, physiologically diffuse disease assessed by pressure-wire pullback has been associated with FFR−/iFR+, while physiological focal disease has been associated with FFR+/iFR−.^12^ We observed a better concordance between FFR and iFR when measures were performed from LCX (87%), compared to LAD (80%). This difference could be explained by the difference in flow between LAD and LCX (ratio 2:1) and by a lesser amount of subtended myocardium in the LCX.^13^ This finding is similar to what was reported in the RESOLVE study that compared FFR and iFR in 1593 lesions with concordance in 80% of cases.^14^ Despite this classification mismatch, 2 large-scale clinical trials, DEFINE-FLAIR and iFR-SWEDEHEART,^15,16^ showed clinical equivalence between FFR and iFR in patients with non-LMCA intermediate stenosis. Lee et al^17,18^ analyzed clinical outcomes in patients with discordant FFR and iFR results, and did not find differences in clinical outcomes when compared to patients with discordant FFR and iFR. The disagreement between FFR and iFR can be explained by differences in hyperemic flow velocity, as described by Cook et al,^19^ who showed also that coronary stenosis classified as FFR+/iFR− had similar coronary flow characteristics to angiographically unobstructed vessels. In any case, physiological indices are a surrogate for the presence of ischemia and, as we have shown, most of the discordance occurs around the gray zone of both FFR and iFR. Besides, it should not be forgotten that FFR has some limitations compared to iFR, given that use of FFR is limited in presence of downstream LAD or LCX lesions, while iFR pullback may allow improved evaluation of tandem lesions.^20,21^ Limitations of FFR also include side effects due to adenosine administration as well as risk of false negative measurements due to failure to induce hyperemia.

### Concordance Between IVUS and Physiology

IVUS MLA has a good correlation with FFR in LMCA lesions, although the optimal cut-off to predict an abnormal FFR value is variable according to the population characteristics. Jasti et al^22^ found that an MLA>5.9 mm^2^ correlated with an FFR>0.75, while Park et al^23^ found an MLA>4.5 mm^2^ in Asiatic population. Regarding iFR, El Hajj et al^24^ recently described a good correlation between an 0.89 iFR cutoff and 6 mm^2^ IVUS MLA. In our study, 192 patients had MLA assessed, and although sensitivity and specificity were good, 29% of patients with FFR+/iFR+ had MLA<6 mm^2^ and 23% of patients with FFR−/iFR− had MLA≥6 mm^2^. In patients with discordance between FFR and iFR, 69% of iFR− patients had an MLA<6 mm^2^ while 58% of iFR+ patients had an MLA≥6 mm^2^.

Interestingly, we have found that an MLA cutoff of 6.0 mm^2^ best predicted an FFR>0.80 and an iFR>0.89, but with relatively low sensitivity, specificity, and predictive accuracy. Jasti et al^22^ described the same MLA cutoff to predict an iFR>0.75 but with higher sensitivity (93%), specificity, (95%) and predictive accuracy (94%). This best performance of FFR could be expected because the 6 mm^2^ cut-off was validated considering FFR as the gold standard.^22^

### Safety of Deferral Based on a Hybrid Decision-Making Strategy Combining iFR and IVUS in Intermediate LMCA Stenoses

Due to the limitations of coronary angiography to assess the severity of intermediate LMCA stenosis, different studies have evaluated intracoronary diagnostic techniques to guide revascularization in these patients. More than 10 years ago, we demonstrated in the LITRO trial that deferring revascularization based on an MLA≥6 mm^2^ was safe.^5^ Regarding FFR, several meta-analyses support its use in LMCA disease.^6,7^ For iFR, however, information is limited to a retrospective observational study with 314 patients, which showed no difference in clinical outcomes when revascularization decision was based on an iFR cutoff ≤0.89.^8^

In terms of practicality, intermediate LMCA stenosis can be evaluated by IVUS, pressure wire, or both. Recently, an expert consensus document from the European Association of Percutaneous Cardiovascular Interventions recommended an approach based on a combination of IVUS and physiology^25^ in intermediate LMCA lesions. Their advice was first do IVUS and, if MLA≥6 mm^2^ defer revascularization, if MLA<4.5 mm^2^ proceed to revascularization, and for MLA 4.5 to 6 mm^2^ decide according to physiology. At this point, it seems reasonable to use either FFR or iFR, given that the evidence we have in the LMCA is similar, if we consider the results of both the DEFINE LM study and what was observed in iLITRO. Our study has shown good clinical outcomes in both groups, without differences in MACE incidence but with a trend to significant less LMCA-related nonfatal myocardial infarction in the deferred group, despite more than half patients had acute coronary syndrome as clinical presentation. Noteworthy, both deferred (50%) and treatment (80%) groups had a high use of IVUS. In the revascularization group, up to 68.6% of patients underwent PCI, with 87.5% use of IVUS to guide revascularization procedure. This strategy could have an impact on outcome, given that patients who underwent CABG showed higher incidence of MACE. This finding is just hypothesis generating, but provides new insight into the safety of PCI for LMCA lesions.

Studies on the clinical impact of physiology in intermediate LMCA lesions have a small number of patients. In this line, the number of patients included in our study is also limited; further studies with a much larger number of patients would be necessary to reach definitive conclusions.

### Study Limitations

The first limitation of this study is its nonrandomized design, but as any all-comers registry, reflects clinical populations seen in real-world clinical practice. Second, patients were enrolled when LMCA stenosis was considered intermediate by the operator, and there was no core lab analysis for coronary angiography, IVUS, or physiology. Nevertheless, again, the study presents patients in routine clinical practice, and this potential bias is reduced by the multicenter design. Third, discordance was identified by differences in functional classification according to a single binary cut point value, but myocardial ischemia is a continuum. Fourth, the number of patients with discordance between FFR and iFR was relatively small, making it difficult, if not impossible, to establish predictors of discordance, but the vast majority of discordant cases were found around the grey zone for both FFR and iFR. Although in our study discordant values of FFR and iFR were assessed using IVUS, other physiological parameters of potential help to explain these discordances (ie, CFR or IMR) were not obtained. Fifth, sample size is relatively small and underpowered for hard clinical outcomes, but our results are in concordance with previous studies for iFR and FFR in LMCA intermediate lesions.^8,26^ Last, the investigators were not blinded to the indices and MLA values, and this might have influenced management in the follow-up; nevertheless, all clinical events were independently adjudicated by the clinical event adjudication committee that was blinded to the anatomic and physiological information.

## Conclusions

In patients with intermediate LMCA stenosis, concordance between FFR and iFR measured from LAD to establish revascularization indication was moderate (80%). In case of discordance, IVUS tended to be more similar to FFR to classify stenosis significance. Deferral of LMCA revascularization based on iFR (combined with IVUS in cases of FFR and iFR discordance) appears to be safe, with similar MACE rate as compared with patients in whom LMCA revascularization was performed.

## ARTICLE INFORMATION

### Sources of Funding

The study sponsor, Fundación EPIC, has received an institutional research grant from Phillips Volcano (the Netherlands) to cover the design and maintenance costs of the electronic CRD. Philips Volcano has had no influence on the study design or protocol in any respect. Philips Volcano is not involved in the conduct of the study, including inclusion, follow-up, data collection, analysis, interpretation of results, drafting, and final approval of the protocol, nor in that of this article. The authors are solely responsible for the design of the study, the writing and editing of the article, and its final content.

### Disclosures

Dr Pérez de Prado has received personal fees from iVascular, Boston Scientific, Terumo, B. Braun‚ and Abbott Vascular. The other authors report no conflicts.

### Supplemental Material

Personal key and participating study sites

Supplemental Methods

Table S1

Figures S1–S2

## Supplementary Material


