# Supplementary material for: Instantaneous Wave-Free Ratio for the Assessment of Intermediate Left Main Coronary Artery Stenosis: Correlations With Fractional Flow Reserve/Intravascular Ultrasound and Prognostic Implications: The iLITRO-EPIC07 Study
Source: Circ Cardiovasc Interv. 2022 Sep 16;15(11):861–71. doi: 10.1161/CIRCINTERVENTIONS.122.012328 (PMC9648986; doi:10.1161/CIRCINTERVENTIONS.122.012328)
Supplement: Supplementary file 1 [file hcv-15-861-s001.pdf]

Instantaneous wave free ratio for the assessment of intermediate left main coronary artery stenosis. Correlations with FFR/IVUS and prognostic implications. The iLITRO - EPIC07 study

Supplementary material

Personal key and participating study sites:

Victoria Vilalta del Olmo, MD (Institut del Cor, Hospital Universitari Germans Trias i Pujol, Badalona, Spain); Gabriela Veiga Fernandez MD PhD (Cardiology Department, Hospital Universitario Marqués de Valdecilla, Santander, Spain); Imanol Otaegui MD PhD (Cardiology Department, Hospital Universitari Vall d'Hebron, Barcelona, Spain); Dolores Vallés MD (Cardiology Department, Hospital Virgen de la Arrixaca, Murcia, Spain); María López-Benito (Cardiology Department, Hospital Universitario de León, León, Spain); Dabit Arzamendi-Aizpurua MD PhD (Cardiology Department, Hospital de la Santa Creu i Sant Pau, Barcelona, Spain) ; Juan José Portero-Portaz MD, Juan Gabriel Córdoba-Soriano MD PhD (Cardiology Department, Complejo Hospitalario Universitario de Albacete, Albacete, Spain); Alberto Pernigotti MD, Mohsen Mohandes MD PhD (Cardiology Department, Hospital Universitari Joan XXIII, Tarragona, Spain); Joan Antoni Gómez-Hospital MD PhD, Montserrat Gracida MD PhD (Cardiology Department, Hospital Universitari de Belvitge, L'Hospital de Llobregat, Spain); Manel Sabate MD PhD, Pablo Vidal MD PhD (Cardiology Department, Hospital Clínic, Institut d'Investigacions Biomèdiques August Pi I Sunyer (IDIBAPS), University of Barcelona, Barcelona, Spain); Fernando Rivero MD PhD, Javier Cuesta MD PhD (Servicio de Cardiología, Hospital de la Princesa, Madrid, Spain); Santiago J. Camacho Freire MD PhD (Cardiology Department, Hospital Universitario Juan Ramon Jiménez,

Huelva, Spain); Neus Salvatella MD (Servei de Cardiologia, Hospital del Mar, Barcelona, Spain); Guisela Flores MD (Division of Interventional Cardiology, Hospital Universitario Reina Sofía, Córdoba, Spain); José Ramón Ruiz Arroyo MD (Cardiology Department, Hospital Clínico Universitario Lozano Blesa, Zaragoza, Spain); Joana Melero Lloret MD (Cardiology Department, Hospital General Universitario de Castellón, Castellón de la Plana, Spain); Sara Casquero-Dominguez MD PhD (Cardiology Department, Hospital Universitario de Puerto Real, Puerto Real, Spain); Ramiro Trillo Nouche, MD (Cardiology Department, Hospital Clínico Universitario de Santiago, Santiago de Compostela, Spain), CIBERCV; Vicente Alcalde Martínez MD PhD (Cardiology Department, Hospital Universitario Virgen de las Nieves, Granada, Spain); Bruno Limpo Bengla MD (Cardiology Department, Hospital de Mérida, Extremadura, Spain); Nieves Gonzalo MD PhD (Servicio de Cardiología, Hospital Clínico San Carlos, Madrid, Spain); José María Hernadez, MD PhD; Erika Muñoz García, MD PhD (Hospital Virgen de la Victoria, Málaga, Spain); Pablo Avanzas, MD PhD; Isaac Pascual, MD (Cardiology Department, Hospital Central de Asturias, Oviedo, Spain); Mario Sadaba, MD PhD; Jose Ramon Rumoroso, MD PhD (Servicio de Cardiología, Hospital Universitario de Galdakao, Galdakao, Spain); Miren Telleria, MD, Irene Elizondo Rua MD (Cardiology Department, Hospital Universitario de Donostia, San Sebastian, Spain); José Antonio Baz, MD PhD, Víctor Alfonso Jiménez Díaz (Cardiology Department, Hospital Alvaro Cunqueiro, Vigo, Spain); Martí Puigfel Pont, MD PhD, Joan Bassaganyas Vilarrasa MD (Cardiology Department, Hospital Universitari Josep Trueta de Girona, Girona, Spain); Felipe Hernandez, MD PhD, Miguel Artaiz Urdaci MD PhD (Cardiology Department, Clínica Universitaria de Navarra, Madrid, Spain); Gema Miñana MD PhD, Agustin Fernandez-Cisnal, MD PhD (Cardiology Department, Hospital Clínico de Valencia, Valencia, Spain); Beatriz

Toledano, MD; Juan Francisco Muñoz, MD (Interventional Cardiology Department, Hospital Universitario Mutua de Terrassa, Terrassa, Spain); Luís Manuel Hernando Romero MD, José Moreu, MD PhD (Cardiology Department, Hospital Universitario de Toledo, Toledo, Spain); Iñigo Lozano, MD PhD; Juan Rondan MD PhD (Servicio de Cardiología, Hospital Universitario de Cabueñes, Gijón, Spain); Fernando Lozano, MD PhD, Ignacio Sanchez Perez MD (Cardiology Department, Hospital General Universitario de Ciudad Real, Ciudad Real, Spain)

Supplementary methods

Inclusion criteria

- Patients with intermediate LMCA lesion (25% to 60% angiographic diameter stenosis on visual estimations) eligible for a pressure guidewire study to determine the iFR
- Patients aged ≥ 18 years
- Patients capable of giving their informed consent

Exclusion criteria

- Patients with an indication for coronary artery bypass graft regardless of the significance of the LMCA lesion
- Patients with LMCA lesions showing ulceration, dissection or thrombus
- Patients with lesions in a previously non-dysfunctional arterial or venous graft in the territory irrigated by the LMCA (protected LMCA)
- Patients with acute coronary syndrome with potentially culprit lesion in the LMCA
- Patients incapable of giving their informed consent

Decision-making algorithm based on FFR and iFR results

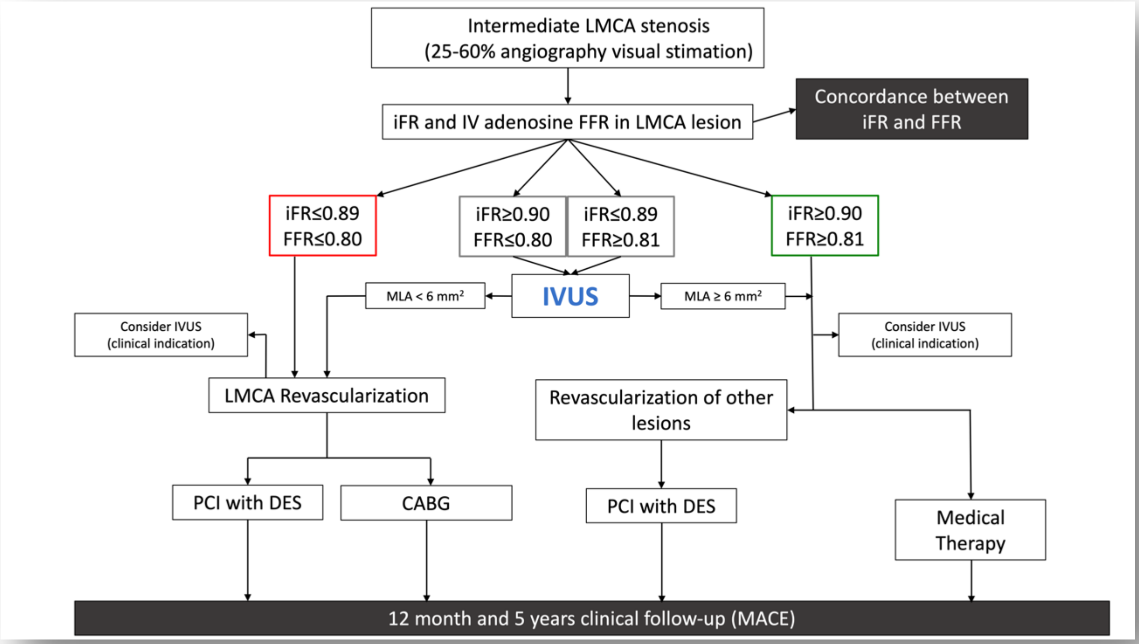

Protocol to perform a study using a pressure guidewire

The patient is eligible for functional assessment in the presence of intermediate LMCA stenoses with visual estimations on the coronary angiography between 25% and 60%. After catheterization using a guide catheter, at least, 200 µg of intracoronary nitroglycerin should be administered to keep coronary reactivity under control. Afterwards, the intracoronary guidewire should be advanced with the sensor placed in the ostium of the guide catheter; also, pressure curves should be brought back to normal for 5 to 10 heart beats. If the lesion has an ostial location, normalization will occur by removing the guide catheter from the coronary artery and placing the guidewire into the aorta. Afterwards, the guidewire should be removed from the catheter, and coronary catheterization performed to advance the guidewire.

The pressure wire should be advanced until, at least, 3 times the diameter of the vessel beyond the most distal stenosis to be able to measure the iFR according to the standard protocol. After measuring the iFR, the guidewire should be removed with pressure curve monitorization until the inside of the guide catheter. At this point, the presence of the pressure calibration loss phenomenon (pressure drift) should be discarded. In case of overt drift (Pd/Pa measured on the catheter tip < 0.98 or > 1.02) measures should be taken again. Afterwards, FFR will be determined during hyperemia through the administration of adenosine in continuous IV infusion at doses ≥ 140 µg/kg/min for, at least, 2 minutes or an IV bolus of 0.4 mg of regadenoson. After measuring FFR, the guidewire should be removed with pressure curve monitorization until the inside of the guide catheter. At this point, the presence of pressure drift should be discarded. In case of overt drift (Pd/Pa measured on the catheter tip < 0.98 or > 1.02) measures should be taken again.

In case of discrepancy between the results of FFR and iFR (FFR ≤ 0.80 with iFR ≥ 0.90 or FFR ≥ 0.81 with iFR ≤ 0.89) IVUS will be performed, and the MLA determined. Revascularization will be indicated with MLAs < 6 mm2 based on the results from the LITRO trial.

Protocol to conduct IVUS studies

IVUS studies will be mandatory if FFR and iFR disagree. In patients eligible for percutaneous treatment of their LMCA lesions, the IVUS is highly recommended to guide the procedure. In the remaining patients (when iFR-guided medical therapy or surgical revascularization is decided) the IVUS is recommended to establish the correlation between the iFR value and the MLA measured on the LMCA whenever possible. The IVUS system used can be mechanical or rotational with resolutions between 20 MHz and 60 MHz.

An 0.014 in intracoronary guidewire will be advanced to perform the IVUS study (it can be the same pressure guidewire used to determine the iFR) towards the left anterior descending or left circumflex coronary arteries. After the administration of 200 µg of intracoronary nitroglycerin, the IVUS catheter will be advanced distal to the LMCA bifurcation. Afterwards, the catheter will be manual or automatically removed until the ascending aorta. It is essential that the guide catheter should remain outside the coronary artery to study the left main coronary artery entirely including its ostial region. The catheter will be placed in the left anterior descending coronary artery (preferably) or left circumflex artery or both (to conduct 2 studies with MLA determination from these positions and eventually pick the one with the lowest values).

In cases of catheter backward jump, even on manual mode (with calcified angulation) it is recommended to move the catheter forward from the aorta to acquire images of the region of interest that had not been properly assessed.

Technical aspects of the assessment of left main coronary artery lesions through fractional flow reserve

The study of LMCA lesions using pressure wire has some particularities that should be addressed when conducting the study:

Location of the lesion

A total of 3 different possible lesion locations can be anatomically distinguished on the LMCA depending on whether lesion location is in the ostium, body or distal portion (bifurcation). The location of the lesion has implications when conducting the study with the pressure wire. When the lesion is found in the ostium or the body, catheterization should be coaxial. Non-coaxial catheterization involves contact of the catheter lumen with the vessel wall to the extent that it can dampen the aortic pressure and artificially elevate the value of the FFR. For this reason, non-selective catheterization is advised when equalizing or normalizing the catheter and guidewire pressures when the latter is placed distal to the lesion to measure the FFR during maximum hyperemia. When the lesion is found in the LMCA distal portion and there is damage to its origin and main branches, both the distal LMCA and each one of its branches should be treated as 1 functional unit regardless of the degree of damage to these branches. To estimate the FFR, measurements are taken from the left anterior descending and left circumflex coronary arteries. The LMCA lesion is considered functionally significantly when the measurements of either one of the 2 main vessels is < 0.80.

Induction of hyperemia

In the assessment of LMCA lesions the use of an intracoronary bolus of adenosine is not recommended because, since the non-selective catheterization of the left coronary artery is required, part of the drugs administered never reach this coronary artery, which is why the induction of hyperemia can be suboptimal. For this reason, the IV administration of drugs whether adenosine (infusions of 140 µg/ kg/min for, at least, 2 minutes) or regadenoson (doses of 0.4 mg in IV bolus) is advised.

Presence of left anterior descending or left circumflex coronary artery lesions

The presence of 1 isolated LMCA lesion is not rare. A series of all-comers treated with diagnostic coronary angiography proved that, in patients with damage to the LMCA, only 9% had 1 single LMCA lesion, 17% had 1 LMCA lesion plus damage to 1 vessel, 35% had 1 LMCA lesion plus damage to 2 vessels, and 38% had LMCA disease plus damage to 3 vessels.

Table S1. Patients in which protocol recommendations to perform or defer LMCA lesion revascularization were not followed.

**Patients with LMCA revascularization in disagreement with protocol recommendations (N=15)**	
FFR- and iFR- (in 2 cases because complicated LMCA lesion in IVUS)	N=9
iFR+ but FFR- and MLA-	N=2
FFR+ but iFR- and MLA-	N=2
iFR+ and FFR- without IVUS study	N=2
**Patients with LMCA revascularization deferral in disagreement with protocol recommendations (N=14)**	
FFR+ and iFR+ but MLA-	N=5
FFR+, iFR+ and MLA+ (1 patient refused revascularization)	N=2
iFR+ and MLA+ without FFR measurement	N=1
iFR+ and MLA+ but FFR-	N=1
iFR+ and FFR- without MLA evaluation	N=1
FFR+ and MLA+ but iFR-	N=2
FFR+ but iFR- without MLA evaluation	N=2

Footnote: in all cases but one who had IFR+, FFR+ and MLA+ but refused revascularization, management decision was according to local heart team recommendation.

FFR+: FFR≤0.80; FFR-: FFR>0.80; iFR+:iFR≤0.89; iFR-: iFR>0.89; MLA+ MLA<6 mm^2; MLA-: MLA≥6 mm^2.

Figure S1. FFR and iFR correlation considering the grey zone for both indices (0.75-0.80 for FFR and 0.86-0.92 for iFR)

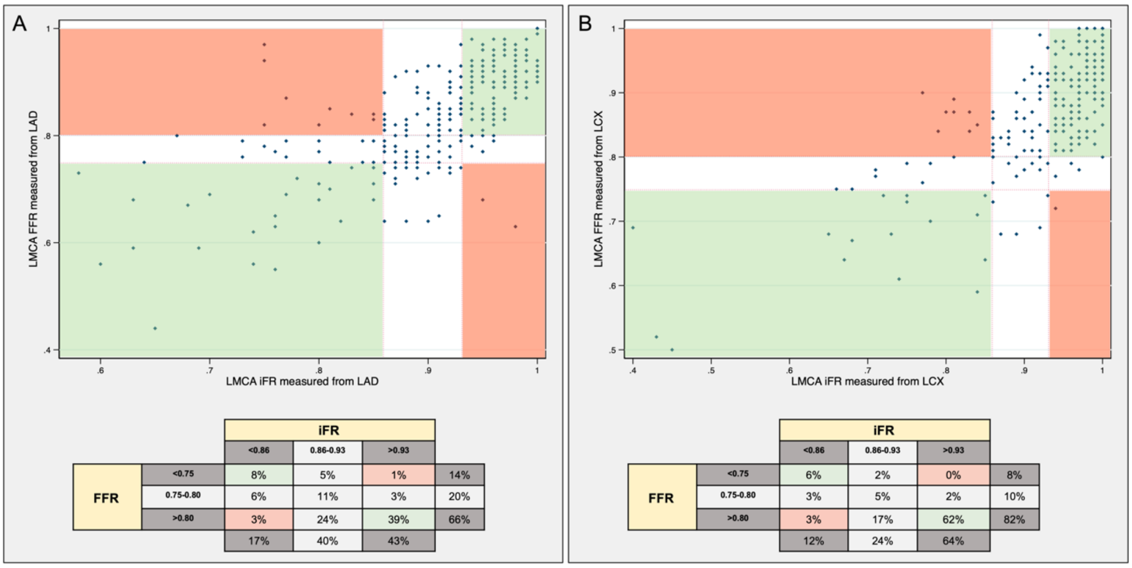

A:

		iFR			
		<0.86	0.86-0.93	>0.93	
FFR	<0.75	8%	5%	1%	14%
	0.75-0.80	6%	11%	3%	20%
	>0.80	3%	24%	39%	66%
		17%	40%	43%	

B:

		iFR			
		<0.86	0.86-0.93	>0.93	
FFR	<0.75	6%	2%	0%	8%
	0.75-0.80	3%	5%	2%	10%
	>0.80	3%	17%	62%	82%
		12%	24%	64%	

<u>Figure caption</u>: A: measurements from LAD; B: measurements from LCX. LMCA: left main coronary artery; LAD: left anterior descendent coronary artery; LCX: left circumflex coronary artery; FFR: fractional flow reserve; iFR: instantaneous wave-free ratio.

Figure S2. Sensitivity and specificity curves on ischemic cut point of FFR, iFR and IVUS parameters.

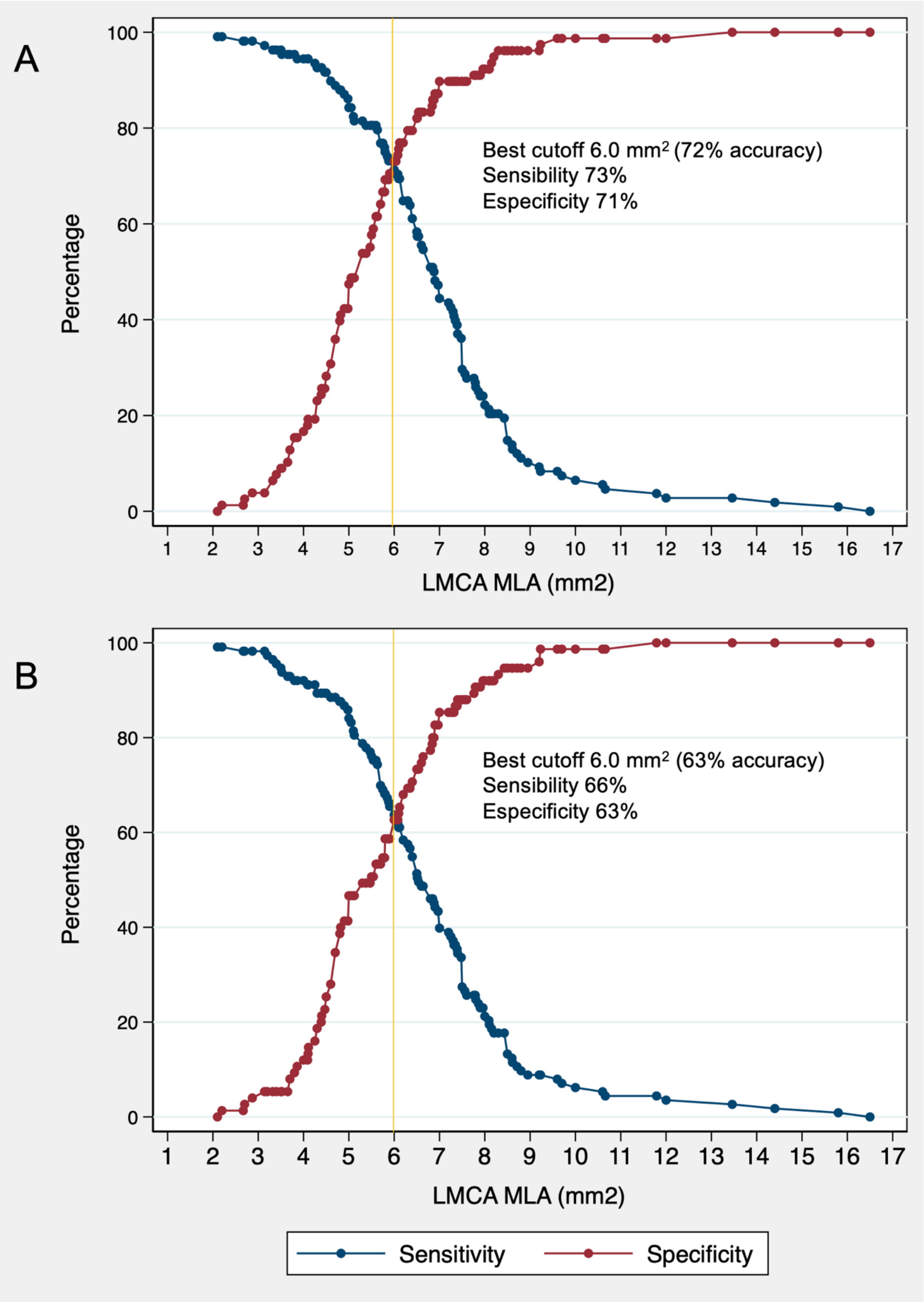

Figure caption: A: FFR vs IVUS; B: iFR vs IVUS.

LMCA: left main coronary artery. MLA: minimal lumen area.
